# Advances and Challenges in the Labor Markets for Healthcare Professionals

**DOI:** 10.3390/healthcare12121233

**Published:** 2024-06-20

**Authors:** Wim Groot, Frits van Merode

**Affiliations:** 1 Maastricht Graduate School of Governance, Maastricht University, Boschstraat 24, 6211 AX Maastricht, The Netherlands; 2 Care and Public Health Research Institute, Maastricht University, 6200 MD Maastricht, The Netherlands; 3 Maastricht University Medical Centre+, 6229 HX Maastricht, The Netherlands

A lack of nurses is a prevalent and persistent problem in many countries [1,2,3,4]. The articles collected in this Special Issue have evidenced this. The nurse shortage is a major barrier to timely access to care for patients. At first glance, this shortage is the result of a demographic change. A continued low birth rate causes an aging population in which demand for (elderly) care outpaces the supply of young workers entering the healthcare labor market. If this is the major explanation, then the nurse shortage is not likely to disappear soon, i.e., in most countries, the birth rate is declining rather than increasing. As long as the birth rate remains below the reproduction rate of two, this aging problem will remain. Without further measures, this would mean that the barriers to access due to a shortage of nurses will continue to exist. We would then have to become accustomed to waiting lists and other barriers to access to health care due to labor shortages.

In many countries, also in rich countries, nurse shortages are concentrated in rural areas because of the unattractiveness of working there [5]. In metropolitan areas, problems occur because of a high turnover of nurses as there are more possibilities to choose another employer [6,7]. This leads to high turnover costs for recruiting and retaining nurses. In metropolitan areas, nurses also often have ample possibilities to find a job outside the healthcare industry. The problem of shortages of nurses cannot be seen in isolation of the mobility of nurses. Several, sometimes conflicting trends are observed. Migration of nurses from poor countries to rich countries has existed for decades but is sensitive to economic shocks. As shown in [8], economic crises in origin countries push more nurse migration. However, recessions in destination countries also lead to redirection of destination countries [9]. Contrary to global trends, the mobility within countries is often very limited, making the labor market for nurses primarily a very local market [10,11].

If we take a closer look, however, it seems there is more at stake. The aging of the population is affecting the entire labor market, not just the labor market of nurses. In addition, although many industries have to deal with a tight labor market and have vacancies, the situation seems more dire for nurses. Access to health care may be more important than timely access to other goods and services, and this makes the nurse shortage more acute. However, in many countries, the shortage of nurses is more severe than the shortage of physicians [1]. Other industries that face an increase in demand due to the aging population also seem to be better able to cope with that. For example, the elderly spend more on leisure activities, but still, the shortage of leisure industry workers seems to be less of a problem than the nurse shortage. Added to that, in many countries, the service industry is growing rapidly. This increases the competition for scarce labor and makes especially healthcare vulnerable to shortages. People change jobs, especially when the new job does not require certification and training for a longer period is needed. Industries such as healthcare are relatively closed labor markets; it is easier to leave the sector for another job than enter it. Partly because of the lack of flexibility and adjustment in healthcare organizations, there is a high outflow of nurses from the profession. Many nurses go either to work in another higher-paying industry or to work abroad. These pull factors exacerbate the shortage of nurses. On top of that, adverse working conditions like shift work during the night and the weekend, a lack of autonomy in the work, and increasing workload (partly exacerbated by the shortage of nurses) do push nurses out of the profession [4,12].

In most countries, health care workers, including nurses, are part of the public sector workforce. Public-sector labor markets are typically less flexible than private-sector labor markets. Wages in the public sector less rapidly adjust and are less responsive to market forces. Increases in public health care budgets are frequently a more important determinant of wage increases for nurses than labor shortages.

One reason why workers choose to work in the public sector is that it comes with greater job security and unemployment protection. This greater security comes at a cost. Wages in the public sector contain a job security discount and are frequently lower than for similar private sector jobs. Work in the public sector is attractive when the risk of becoming unemployed is high. It then provides additional insurance against becoming unemployed, but in a tight labor market this becomes a disadvantage. In a labor market characterized by low unemployment and a large number of vacancies, industries in which wages can quickly be adjusted to attract and retain workers are at an advantage. As wages for nurses are frequently lower than those of similar positions in the private sector and cannot be easily adjusted because wage increases require increased public funding for health care, this puts the sector at a disadvantage. Consequently, the labor market for nurses seems ill adjusted to the demographic change that leads to more demand and less supply of health care workers.

Even if wages and labor conditions for nurses are improved and the hidden reservoir of nurses has been activated, a shortage of nurses may prevail. More is needed than recruiting workers for the profession. The labor productivity of nurses needs to improve, so the increased demand for health care can be met by a (relatively) lower number of nurses. This does not mean that nurses need to work harder, but rather that new healthcare operations models are developed, i.e., nursing work can be automated, deskilled, and transferred to non-nurse staff. All these activities can also make nursing work more attractive. Important is also that decision autonomy, which is essential for professional work, is regulated and cultivated. This makes work for nurses more attractive. This is a task at all levels of the healthcare system. Overall, more investments in nurse labor-saving innovations in health care are needed.

During the last century in many countries, informal care has been transferred to professional care providers, of which nurses have taken a big part. Now we have to transfer some nursing activities back to informal caregivers and patients. It also requires a demedicalization of our society to lower the demand for professional care. 

Unfortunately, the health care industry lags behind other industries in increasing labor productivity [13]. This is partly due to the labor intensity of the profession and partly because of the prevailing idea that health care should be provided hands-on. On top of that, the public sector nature of the industry eliminates the competitive incentive to increase labor productivity. For publicly funded health care organizations, it is frequently easier and less risky to reduce the amount of care they deliver when faced by labor shortages than to invest in labor-saving technologies. The innovation of healthcare operations models is going too slowly.

A major problem in both policies and research is the focus on specific aspects of the nurse shortage problem and approaches to reduce these shortages. There have been policies to increase wages of nurses and other measures to attract and retain nurses in the profession. There have been policies to increase labor productivity and to reduce demand for professional care. What is missing is an integrated approach. This also pertains to research on this topic. What is lacking is an integrated analysis of the relative contribution of all of these (and other) interventions to reducing the labor shortage of nurses.
